# Rosuvastatin improves impaired endothelial function, lowers high sensitivity CRP, complement and immuncomplex production in patients with systemic sclerosis - a prospective case-series study

**DOI:** 10.1186/ar4285

**Published:** 2013-09-04

**Authors:** Orsolya Timár, Zoltán Szekanecz, György Kerekes, Judit Végh, Anna V Oláh, Gábor Nagy, Zoltán Csiki, Katalin Dankó, Szilvia Szamosi, Ágnes Németh, Pál Soltész, Gabriella Szücs

**Affiliations:** 1 Departments of Clinical Immunology and Angiology, Institute of Medicine, University of Debrecen Medical and Health Science Center, Móricz str 22, Debrecen, 4032, Hungary; 2 Department of Rheumatology, Institute of Medicine, University of Debrecen Medical and Health Science Center, Nagyerdei str 98, Debrecen, 4032, Hungary; 3 Department of Laboratory Medicine, University of Debrecen Medical and Health Science Center, Nagyerdei str 98, Debrecen, 4032, Hungary

**Keywords:** rosuvastatin, systemic sclerosis, atherosclerosis, cardiovascular, endothelial function, flow-mediated vasodilation, arterial stiffness, pulse-wave velocity

## Abstract

**Introduction:**

We studied the effect of rosuvastatin on endothelial and macrovascular function, cardiovascular risk factors and the complement pathway in patients with systemic sclerosis (SSc).

**Methods:**

Altogether 28 patients with SSc underwent laboratory and complex vascular assessments before and after six months of 20 mg rosuvastatin treatment. Flow-mediated dilation (FMD) of the brachial artery, as well as carotid artery intima-media thickness (ccIMT), carotid-femoral and aorto-femoral pulse wave-velocity (PWV) were analyzed by ECG-synchronized ultrasound. Ankle-brachial index (ABI) was determined by Doppler, and forearm skin microcirculation was assessed by Laser Doppler perfusion monitoring.

**Results:**

Brachial artery FMD significantly improved upon rosuvastatin therapy (2.2% ± 3.3% before versus 5.7% ± 3.9% after treatment, *P *= 0.0002). With regard to patient subsets, FMD significantly improved in the 21 lcSSc patients (from 2.1% to 5.6%, *P *= 0.001). In the seven dcSSc patients, we observed a tendency of improvement in FMD (from 3% to 6%, *P *= 0.25). Changes in PWV, ccIMT and ABI were not significant. Mean triglyceride (1.7 ± 0.97 versus 1.3 ± 0.46 mmol/l, *P *= 0.0004), total cholesterol (5.3 ± 1.6 mmol/l versus 4.2 ± 1.3 mmol/l, *P *= 0.0003), low density lipoprotein cholesterol (3.0 ± 1.3 versus 2.2 ± 1.0 mmol/l, *P *= 0.005) and C-reactive protein levels (CRP) (5.1 ± 5.2 versus 3.4 ± 2.7, *P *= 0.01) levels significantly decreased after rosuvastatin treatment. Mean C3, C4 and IC levels also decreased significantly as compared to pretreatment values.

**Conclusions:**

Six-month rosuvastatin therapy improves endothelial function and lowers CRP, C3, C4 and IC levels indicating possible favourable effects of this statin on the cardiovascular and immune system in SSc.

## Introduction

Systemic sclerosis (SSc) is a systemic autoimmune disease of uncertain etiology characterized by progressive fibrosis of the skin, the small blood vessels and various internal organs. Population-based and other cohort studies have emphasized that survival is decreased in patients with SSc [1-3]. Several reports including our previous studies, have found macrovascular abnormalities in SSc patients. These include endothelial dysfunction indicated by abnormally low flow-mediated dilation (FMD) of the brachial artery [4,5], increased carotid intima-media thickness (IMT) and increased arterial stiffness [6-8]. Statins may improve endothelial dysfunction, arterial stiffness and reduce levels of inflammatory markers in various conditions including chronic kidney disease, rheumatoid arthritis and dyslipidemia. In SSc, the vascular effects of atorvastatin, simvastatin and pravastatin have been investigated so far [9]. Atorvastatin exerted beneficial effects on microvascular function, digital ulcers, and soluble markers of endothelial function, as well as FMD [10,11]. Simvastatin and pravastatin reduced the production of soluble endothelial activation markers [12,13].

It is not fully clear which of the multiple mechanisms of statins may be involved in SSc-related vascular changes. Although a pronounced reduction of LDL-cholesterol is present in patients undergoing statin therapy, additional mechanisms have been suggested to improve endothelium-dependent vasodilation in case of HMG-coA reductase inhibitor therapy. A key point is promoting activity of the endothelial NO synthase. This can either be caused by a weakened interaction between the endothelial NO synthase (eNOS) and caveolin-1, or the association of e-NOS and hsp90, as well as by an upregulation of eNOS mRNA by inhibition of the Rho kinase pathway or a reduction in ICAM-1 and P-selectin levels, which also result in increased endothelial NO production [14].

In this study, we wished to determine the effects of rosuvastatin, a potent reducer of total (TC) and LDL cholesterol (LDL-C) [15,16], on serum inflammatory markers and complement levels, on endothelial and macrovascular function, as well as on arterial stiffness in patients with SSc. In addition, we determined the possible effects of rosuvastatin on microvascular function by assessing cutaneous blood flow. The primary endpoint of the study was rosuvastatin effects on the vasculature (FMD, carotid artery intima media thickness (ccIMT), pulse wave velocity (PWV), ankle-brachial index (ABI)), while the secondary outcome included laboratory marker changes (lipids, C-reactive protein (CRP), immune complexes and complement). The assessment of acute phase reactants and complement has been included in scleroderma activity index [17].

## Methods

### Patients

SSc patients undergoing follow-up visits at our institution were randomly screened for inclusion and exclusion criteria described below. Altogether 28 patients including 25 women and three men were eligible for the study. The diagnosis was established according to the 1980 American College of Rhematology criteria for SSc [18]. The mean age of the patients was 60.4 ± 11.0 years (range: 34 to 83 years) and the mean disease duration was 13.6 ± 7.7 years (range 2 to 30 years). Among these patients, 21 (75%) had the limited (lcSSc) and seven (25%) had the diffuse cutaneous form of SSc (dcSSc). Clinical manifestations of SSc included Raynaud's phenomenon (96%), distal skin manifestations including sclerosis and ulcers (68%), proximal skin involvement (25%), cardiac manifestations including conduction defects, atrial fibrillation, arrhythmias (25%), gastrointestinal manifestations, such as esophageal dysmotility, gastroesophageal reflux (54%), renal involvement (4%) and sicca syndrome (10%). Six patients (22%) had an overlap syndrome with another autoimmune disease: two patients had poly-dermatomyositis, one had systemic lupus erythematosus and three had rheumatoid arthritis in addition to SSc. The patients' medications are listed in Table 1. All recruited patients were non-smokers and their mean body mass index (BMI) was 23.1 ± 4.1 kg/m^2^.

**Table 1:** Pharmacological therapy of SSc patients at inclusion

Medication	Number of patients	%
ARB/ACE inhibitors	21	75
Calcium channel blockers	16	57
Beta blockers	8	29
Corticosteroids	9	32
Pentoxifylline	23	82
Nitroglycerine	4	14
Vitamin E	8	29
Platelet Aggregation Inhibitors	16	57
H2-receptor blockers or proton-pump inhibitors	14	50
Immunosuppressive agents	3	11
NSAIDs	5	18
Bisphosphonates	3	11
Others (diuretics, tramadol, benzodiazepines, bronchodilators)	14	50

NSAIDs, non-steroidal anti inflammatory drugs.

Patients were included in the study if lcSSc or dcSSc was present, and the patients exhibited microvascular symptoms including new digital ulcers and active Raynaud's symptoms despite ongoing therapy. Patients had not been on any lipid lowering drug therapy for the six months prior to this study.

Exclusion criteria included hyperglycemia, acute systemic infection, uncontrolled hypertension, carotid sinus hyperesthesia, permanent atrial fibrillation, an ejection fraction (EF) less than 50% as determined by echocardiography, severe pulmonary arterial hypertension (PAH), active ulcers at any of the measurement sites or lack of patient's informed consent.

Approximately 60 patients were screened for the study and 28 patients meeting the inclusion criteria were enroled. Any patients meeting any exclusion criteria, those on vasoactive drugs, such as prostanoids, or patients undergoing frequent treatment modifications, were excluded.

Written informed consent was obtained from all subjects. The study was performed according to the Declaration of Helsinki under the auspices of the University of Debrecen Research Ethics Committee and the ETT-TUKEB central Ethics Committee (No. 14804-1/2011-1018EKU).

### Study protocol

Both laboratory analyses and the clinical examinations described below were performed on two occasions, directly before and after the rosuvastatin treatment period. Each patient recieved 20 mg rosuvastatin daily for six months. All patients tolerated the drug well and all of them could complete the study. On the day of vascular assessments, blood samples were drawn between 7 a.m. and 8 a.m. after an eight-hour fasting period. Samples were stored at room temperature and analyses were performed within two hours. Von Willebrand factor antigen (vWF) samples were stored on ice until analysis. Before vascular assessments, the use of vasoactive and antioxidant drugs, as well as alcohol or caffeine consumption, was suspended for 24 hours. Examinations were performed under standardized conditions after a 10-minute resting period in a recumbent position. Vascular assessments were carried out in a quiet, darkened study room with a temperature of 22 ± 1°C according to the recommendations of Laurent *et al*. [19]. All assessments were performed by a single observer (OT)

### Vascular assessments

Brachial artery FMD assessment was performed by the same skilled operator according to the methodology described by Corretti *et al*. [20] under standardized conditions [21] using a 10 MHz linear array transducer of a HP Sonos 5500 (Hewlett Packard) ultrasound equipment. Briefly, a longitudinal section of the brachial artery of supine patients 4 to 7 cms proximal from the antecubital fossa was obtained at end-diastole (upon R wave of ECG) and the arterial diameter (BAD_basal_), namely the distance between the proximal and distal media-adventitia borders, was measured. Results of five repeated measurements were averaged. After 4.5 minutes of distal (forearm) blood pressure cuff occlusion with 50 mmHg suprasystolic pressure, reactive hyperaemia-induced maximal diameters (BAD_max_) were measured within three minutes after release. Images were digitized for further documentation. FMD was calculated as [(BAD_max_-BAD _basal_)/BAD_basal_] × 100 and expressed in % compared to the basal diameter. The intraobserver variability of FMD was excellent: the calculated coefficient of variation (CV) and intraclass correlation coefficient (ICC) were 5% and 0.935, respectively, indicating very good reproducibility.

Aorto-femoral pulse wave velocity (c-fPWV) was measured as previously described elsewhere [22] on a HP Sonos 5500 ultrasound equipment with simultaneous ECG recording. Carotid-femoral pulse wave velocity (c-fPWV) was measured during the same examination with minor changes compared to the a-f PWV assessment. C-f PWV was determined between the left common carotid artery, 1 cm proximal to the bifurcation and the right common femoral artery at the level of the inguinal ligament using the linear array transducer. The distance between the two sites was defined as the difference between the sternal jugulum - carotid measurement point distance and the sternal jugulum-femoral measurement point distance. C-f PWV was again the quotient of the distance and the transit time, the time difference between the foot-to-foot intervals gained at the two measurement sites [23-25].

Common carotid artery intima-media thickness (ccIMT) was measured according to current guidelines [26] by the same examiner with the HP Sonos 5500 ultrasound equipment described above. Briefly, longitudinal B-mode views of the common carotid arteries were taken by medio-lateral probe position, then high magnification images were frozen at end-diastole. ccIMT, defined as the distance between the first and second echogenic lines corresponding to the intima-lumen and media-adventitia interfaces, respectively, was measured offline 1 cm from the carotid bulb or the nearest plaque-free segment on the far wall according to the leading edge principle. Ten measurements were performed and averaged on either side and the value of ccIMT was expressed in millimeters. The intraobserver variability of ccIMT was excellent: the calculated CV and ICC were 4.2% and 0.98, respectively, indicating very good reproducibility.

Assessment of ABI was carried out according to TASC II. guidelines [27]. A 10 to 12 cm sphygmomanometer cuff was placed just above the ankle and a handheld CW Doppler instrument (Vasodop 8 MHz, MediCAD Ltd, Miskolc, Hungary) was used to measure the systolic pressure of the posterior tibial and dorsal pedal artery of each leg. The higher of these pressures was divided by the higher brachial systolic blood pressure value to form the ABI.

Microvascular skin perfusion was assessed by Laser Doppler (LD) perfusion monitoring [28]. During this examination, a 780 nm wavelength laser beam penetrates the skin to a depth of 1 to 1.5 mm and a fraction of the light is scattered back by moving blood cells resulting in a frequency shift according to the Doppler principle. Therefore, a signal proportional to tissue perfusion is generated. Provocation tests, such as heat, postocclusive reactive hyperemia (PORH) or biochemical agents applied by iontophoresis allow for testing skin reactivity. We applied a standard laser probe (PF 408) fixed in a straight probe holder (PH 08) of a Periflux PF 4001 LD flowmeter (Perimed AB, Järfällä, Sweden). The LD apparatus was connected to a laptop, which displayed recordings and saved the information for further offline analysis by the Perisoft for Windows (Ver. 2.5.5) software (Perimed AB). Measurements were carried out with the patients in a supine position. The probes were placed on the volar side of the mid-forearm skin, avoiding superficial subcutaneus blood vessels, and basal blood flow was recorded for eight minutes. Afterwards, during continuous recording of skin blood flow, the upper arm was obstructed for a three minute period by a 10 to 12 cm sphygmomanometer cuff inflated to 50 mmHg suprasystolic pressure, then cuff was suddenly deflated and forearm skin blood flow was further recorded for five minutes. During this period, blood flow returned to baseline. Blood flow (basal, peak, biological zero) was expressed in arbitrary perfusion units (PU) and relative changes compared to basal values were assessed, time to half before hyperaemia (TH1, seconds), time to maximum flow (TM, seconds) and time to half recovery (TH2, seconds) were analysed, and the occlusion areas (AO), hyperaemic areas under curve (AH, PU*seconds), as well as hyperemia repayment (AH/AO) were determined. In addition, we assessed the slope of the curve as it reached maximum perfusion after cuff release (acceleration slope) and upon return to basal skin flow (deceleration slope).

### Laboratory analyses

Serum biochemical markers and high sensitivity CRP (hsCRP) were analysed on a Modular P-800 analyser (Roche Ltd, Mannheim, Germany). Serum total cholesterol, triglyceride and uric acid levels were determined by enzymatic colorimetric assay, HDL and LDL- cholesterol were analysed by homogenous enzymatic assay. Serum glucose and urea levels were measured using enzyme kinetic UV assay, serum creatinine was determined by the compensated Jaffe kinetic method. Estimated glomerular filtration rate (GFR) was calculated from the serum creatinine by the MDRD 175 (Modification of Diet in Renal Disease study group) formula. HsCRP was assessed by wide range immunoturbidimetric assay, hsCRP levels >5 mg/L were considered elevated.

Plasma levels of circulating vWF, a marker of endothelial cell activation, were determined by STA Liatest vWF immunoturbidimetric assay using microlatex particles coated with polyclonal rabbit anti-human vWF antibodies (Diagnostica Stago, Asnieres, France). After mixing the reagent with plasma, the degree of agglutination is proportional to the amount of vWF present in the plasma sample. The reference range for the test is 50% to 160%.

Hematological parameters including hemoglobin (Hgb), white blood cell and platelet counts were determined using a Sysmex XE-2100D automated haematology analyzer (Sysmex Corp., Kobe, Japan). Erythrocyte sedimentation rate (ESR) was determined by the Westergren method.

Circulating immune complexes (IC) were detected by polyethylene glycol precipitation method. Serum complement C3 and C4 levels were measured by nephelometry on a Siemens-Dade-Behring BN-II nephelometer. Laboratory reference ranges were 0.9 to 1.8 g/L for C3, 0.1 to 0.4 g/L for C4 and an extinction of 0 to 170 for IC.

### Statistical analysis

Due to the nature of the study (before/after comparison), paired one-tailed/two-tailed *t*-tests were used for statistical evaluation. *P *values < 0.05 were considered significant. Data represent a normal distribution, as shown by the Kolmogorov-Smirnov test. Correlations were assessed using SPSS software version 11.0. The Pearson correlation coefficients were determined and r values at the *P *< 0.05 level were considered significant.

## Results

### Effects of rosuvastatin on micro-and macrovascular function

FMD significantly improved after six months of rosuvastatin therapy (2.3% ± 3.3% before versus 5.7% ± 3.9% after treatment, *P *= 0.0002) (Table 2). Altogether 23 patients responded with an increase in occlusion-provoked vasodilation of the brachial artery following rosuvastatin treatment.

**Table 2:** Vascular assessments before and after rosuvastatin treatment in SSc patients

Parameter	Pre-treatment mean (S.D.)	Post-treatment mean (S.D.)	*P *value^a^
FMD (%)	2.3 (3.3)	5.7 (3.9)	**0.0002**
Right ccIMT (mm)	0.675 (0.144)	0.681 (0.142)	ns (0.38)
Left ccIMT (mm)	0.717 (0.172)	0.701 (0.165)	ns (0.3)
Carotid-femoral PWV (m/s)	8.7 (2.6)	8.1 (1.9)	ns (0.1)
Aorto-femoral PWV (m/s)	8.8 (2.2)	8.3 (2.1)	ns (0.15)
Right Ankle-Brachial Index	1.1 (0.16)	1.1 (0.27)	ns (0.4)
Left Ankle-Brachial Index	1.1 (0.14)	1.1 (0.19)	ns (0.4)
Laser Doppler acceleration slope (U/s)	14.6 (14.8)	10.0 (10.3)	ns (0.08)
Laser Doppler deceleration slope (U/s)	-1.13 (0.92)	-0.64 (1.09)	**0.021**

^a^Significant differences are indicated in bold. ccIMT, common carotid intima-media thickness; FMD, flow-mediated vasodilation; ns, non significant; PWV, pulse wave velocity; SSc, systemic sclerosis.

With regard to patient subsets, FMD significantly improved in the 21 lcSSc patients, from 2.1% to 5.6% (*P *= 0.001). In the seven dcSSc patients, we observed a tendency of improvement in FMD, from 3% to 6% (*P *= 0.25). The non-significant change in dcSSc may be the result of low patient number (data not shown).

In 11 of the 28 patients (39.3%), baseline carotid-femoral PWV (c-fPWV) values were above the average reference values of age-, lipid- and blood-pressure-status-matched European patients [29] (Table 2). Neither aorto-femoral nor carotid-femoral PWV showed significant improvement upon rosuvastatin treatment (a-f PWV: 8.8 ± 2.2 m/s before versus 8.3 ± 2.1 m/s after therapy, *P *= 0.15; c-f PWV: 8.7 ± 2.6 m/s before versus 8.1 ± 1.9 m/s after treatment, *P *= 0.1) (Table 2). However, by the end of rosuvastatin treatment, only 5/28 patients (17.9%) had c-fPWV above the mentioned reference values.

The mean ABI, indicator of peripheral arterial disease, was 1.1 ± 0.2 on both sides and remained unchanged after rosuvastatin therapy (Table 2).

Ultrasound analysis of the common carotid arteries revealed a mean ccIMT of 0.68 ± 0.14 mm on the right and 0.72 ± 0.17 mm on the left side at baseline. After rosuvastatin therapy, these values were 0.68 ± 0.14 mm (*P *= 0.38) and 0.70 ± 0.17 mm (*P *= 0.3), respectively (Table 2). Thus, statin treatment did not result in any improvement in carotid atherosclerosis.

Laser Doppler analysis of the forearm skin flow during PORH testing revealed decreases in the acceleration and deceleration slope of the curves following rosuvastatin therapy compared to pretreatment values (acceleration slope: 14.6 ± 14.8 versus 10.0 ± 10.3 U/second, *P *= 0.081; deceleration slope: -1.13 ± 0.92 U/second versus -0.64 ± 1.09 U/second, *P *= 0.021) (Table 2). Neither basal, peak nor biological zero skin perfusion, nor AH nor any of the time characteristics (TM, TH1, TH2) showed significant changes compared to pretreatment values (data not shown).

### Laboratory parameters

The presence of antinuclear autoantibodies (ANA) among patients was the following: 26/28 patients (93%) were ANA positive, 12/28 patients (43%) had antibodies against extractable nuclear antigen (ENA), 1/28 (4%) against nuclear ribonucleoproteins (RNP), none against Smith antigen (Sm), 3/28 patients (11%) against SS-A (Ro) antigen, none against SS-B (La), 12/28 patients (43%) tested positive for antibodies against topoisomerase I (Scl-70), and 2/28 (7%) were positive for antibodies against histidyl-tRNA synthetase (Jo-1).

Baseline serum lipid levels indicated that 10% of patients had hypertriglyceridaemia (TG >2.3 mmol/L), 50% had hypercholesterolemia (total cholesterol >5.2 mmol/lL) and 32% had elevated LDL-C levels (>3.4 mmol/L). At baseline, 11 out of 28 patients (39%) had low HDL-C levels (<1.2/<1.0 mmol/L for women/men, respectively). Reference values were determined as recommended for the medium cardiovascular risk group based on the European SCORE chart [30].

Among blood chemistry values, lipid parameters showed significant improvement after six months of rosuvastatin therapy. Mean TG levels decreased from 1.70 ± 0.97 mmol/L to 1.30 ± 0.46 mmol/L following therapy (*P *= 0.0004). Total cholesterol decreased from 5.3 ± 1.6 mmol/L to 4.2 ± 1.3 mmol/L (*P *= 0.0003), LDL-C levels decreased from 3.0 ± 1.3 mmol/L to 2.2 ± 1.0 mmol/L (*P *= 0.0046), while mean HDL-C levels remained unchanged (1.5 ± 0.8 mmol/L before versus 1.5 ± 0.6 mmol/L after therapy, *P *= 0.33) (Table 3). Non-HDL cholesterol levels also displayed a significant decrease after statin therapy (3.8 ± 1.5 versus 2.5 ± 1.3 mmol/L, *P *= 0.0003.)

**Table 3:** Laboratory parameters before and after rosuvastatin treatment in SSc patients

Parameter	Pre-treatment mean (S.D.)	Post-treatment mean (S.D.)	*P *value^a^
Erythrocyte sedimentation rate (mm/hour)	21 (15.6)	24.7 (19)	ns (0.15)
CRP (mg/L)	5.1 (5.2)	3.4 (2.7)	**0.01**
Glucose (mmol/L)	5.3 (1.0)	5.5 (1.3)	ns (0.24)
Urea (mmol/L)	6.0 (2.4)	5.9 (2.5)	ns (0.5)
Creatinine (μmol/L)	67.5 (19.7)	63 (16.9)	ns (0.062)
GFR (ml/min)	82.1 (14.0)	84.8 (10.8)	ns (0.078)
Triglyceride (mmol/L)	1.7 (0.97)	1.3 (0.46)	**0.0004**
Total cholesterol (mmol/L)	5.3 (1.57)	4.2 (1.28)	**0.0003**
LDL (mmol/L)	3.0 (1.3)	2.2 (1.0)	**0.005**
Non-HDL (mmol/L)	3.8 (1.5)	2.5 (1.3)	**0.0003**
HDL (mmol/L)	1.5 (0.84)	1.5 (0.6)	ns (0.33)
Uric acid (μmol/L)	263 (56)	273 (77)	ns (0.22)
von Willebrand factor (%)	209 (90)	193 (75.6)	ns (0.092)
Hemoglobin (g/L	125 (12.7)	126 (12.5)	ns (0.242)
White blood cell count (10^9^/L)	6.7 (2.6)	7.1 (2.7)	ns (0.191)
Platelet count (10^9^/L)	250 (62)	265 (64.5)	ns (0.064)
Complement 3 (g/L)	1.81 (0.4)	1.62 (0.32)	**0.001**
Complement 4 (g/L)	0.31 (0.13)	0.27 (0.1)	**0.001**
Immune complex (extinction)	183.6 (110)	135.5 (55)	**0.005**

^a^Significant differences are indicated in bold, CRP, C-reactive protein; GFR, glomerular filtration rate; HDL, high-density cholesterol; LDL, low-density cholesterol; ns, non-significant; SSc, systemic sclerosis.

Among acute phase reactants, hsCRP levels showed a significant decrease, from 5.1 ± 5.2 mg/L to 3.4 2.7 mg/L (*P *= 0.01). ESR, renal function tests and full blood counts showed no biologically relevant changes upon statin therapy as compared to baseline values (Table 3).

Baseline circulating vWF antigen levels were abnormally high in 63% of patients and although mean vWF antigen levels showed a slight decrease after rosuvastatin treatment (209 ± 90% versus 193 ± 76%), this change remained statistically insignificant (*P *= 0.09) (Table 3).

Serum immune complex levels (IC) were initially elevated and levels returned to normal after rosuvastatin therapy (extinction: 183.6 versus 135.5, respectively, *P *= 0.007), while C3 (1.81 versus 1.62 g/L) and C4 levels (0.33 versus 0.27 g/L) displayed a significant decrease after rosuvastatin treatment (*P *= 0.001) within the reference range.

There were no significant differences in any laboratory parameters with regard to rosuvastatin treatment in lcSSc versus dcSSc (data not shown).

## Discussion

We have previously described impaired FMD in SSc patients compared to age- and sex-matched controls [5]. Our current pretreatment FMD results in scleroderma patients are in concordance with our previous measurements. The detected significant improvement in endothelial function following six months of rosuvastatin treatment, however, is a novel finding which has not been demonstrated in SSc patients before. Comparing this effect of rosuvastatin to the vascular effects of atorvastatin treatment reported by other investigators [31-34], it is likely that the two statins are similar with respect to their effect on endothelial function (FMD). Whether favourable effects of rosuvastatin on endothelial function are also accompanied by improvement in clinical vascular symptoms such as Raynaud's phenomenon, digital ulceration or Rodnan skin score in SSc has yet to be investigated.

The duration of therapy is a decisive feature of successive statin treatment in SSc. While in one study [33], an eight-week atorvastatin (20 mg) treatment in SSc exhibited no effect on endothelial function as assessed by Laser Doppler imaging, another [34] described beneficial effects of 24-month atorvastatin (10 mg) therapy on Raynaud's phenomenon of SSc patients. Thus, the beneficial effects of statins on the vasculature, as well as on the underlying inflammatory disease may be expected only after six months or more.

As recently reviewed [35], there is an ongoing debate about the extent and presence of atherosclerosis in patients with SSc. By assessing ccIMT, PWV, FMD and ABI we wished to add further data to this debate.

In our present study, the mean pretreatment values of right and left ccIMT were within the 25th and 75th percentile range of the given age group as described in large-scale European cohort studies [36,37]. Our current findings support the results of studies reporting normal ccIMT in scleroderma patients and it seems these studies outnumber those detecting abnormally high ccIMT values [35]. Baseline ABI values were also normal in this study, in accordance with other studies describing either normal or mildly reduced ABI in SSc patients [35]. Regarding changes after six months of rosuvastatin therapy, our assumptions of favourable outcome were based on the results of previously conducted clinical trials with rosuvastatin on nonrheumatic patients and on a few pilot studies with rosuvastatin in patients with rheumatoid arthritis or hyperlipidaemia. Two years of treatment with high-dose rosuvastatin has been shown to result in regression of coronary atherosclerosis, as documented by intravascular ultrasound and quantitative coronary angiography [38,39]. In the METEOR study, 40 mg rosuvastatin resulted in a significant reduction in the rate of progression of maximum ccIMT values over two years in middle-aged patients with subclinical atherosclerosis [40]. Yet, our present findings suggest that six months of rosuvastatin (20 mg) therapy yields no change in mean ccIMT in scleroderma patients. The reasons why we could not detect similar changes as the investigators in the METEOR study could be the lower dose and shorter duration of rosuvastatin therapy.

The frequency of PWV values above mean European reference values [29] decreased from 11/28 to 5/28 following rosuvastatin therapy. Somewhat suprisingly, a-f and c-fPWV were elevated only in 39% of patients in this study and mean values exhibited no statistically significant changes following rosuvastatin therapy. Multiple explanations, including concomittant use of calcium channel blockers and/or ACE-inhibitors (Table 1), the duration of statin therapy may be put forward. In order to interpret our results appropriately, repetition of these examinations with longer treatment periods and comparison with PWV progression in nontreated patients will be required.

We decided to add Laser Doppler measurements to our study protocol because some studies had previously indicated impaired cutaneous vasodilatory response to ischemia in SSc patients [41]. Our Laser Doppler flowmetry measurements revealed significantly slower deceleration slope during PORH testing of the forearm skin after rosuvastatin compared to pretreatment values. Yet, the relevance of these slopes in determining microcirculation has not yet been fully determined.

Among laboratory parameters, in our current study marked reductions in total TG, total and non-HDL cholesterol as well as LDL-C cholesterol were observed after six months of rosuvastatin treatment. HDL-C levels remained unchanged after therapy; however, the frequency of HDL-C levels below normal decreased. The changes in total cholesterol and LDL-levels correspond to expectations based on results of clinical trials conducted previously to assess efficacy of rosuvastatin in comparison with other statins [15,16,42]. A possible explanation for not observing significant changes in HDL-C levels may be the fact that the majority of SSc patients had HDL-C levels within the normal range at baseline.

Among inflammatory markers, hsCRP levels significantly decreased following rosuvastatin treatment. Rosuvastatin reduces CRP levels in low cardiovascular risk individuals in the general population [43]. There is substantial evidence that hsCRP is an independent cardiovascular risk factor in the general population, but its predictive value in early stages of atherosclerosis is doubtful [44,45]. To the best of our knowledge, this is the first study to show that CRP levels improve after six months of 20 mg rosuvastatin therapy in patients with SSc.

Levels of circulating vWF antigen have been found elevated in patients with Raynaud's phenomenon and SSc [46] as a sign of endothelial injury. In our patient cohort, rosuvastatin treatment resulted in a slight, non-significant decrease in elevated circulating vWF levels.

Although the exact pathomechanism of SSc is yet unknown, lately, activation of the complement system has been suggested and immune complex deposition, particularly in the perivascular and subendothelial region, has been described [47]. In our current study, we observed elevated serum immune complex levels returning to normal following rosuvastatin treatment. In addition, C3 and C4 levels showed a significant decrease after rosuvastatin treatment.

## Conclusions

In conclusion, rosuvastatin improves brachial artery FMD, corrects dyslipidemia and decreases CRP, complement 3 and 4 and immune complex levels, while it may not reduce arterial stiffness or carotid atherosclerosis following a six-month treatment period in SSc patients with intermediate cardiovascular risk. More long-term studies carried out in larger patient cohorts are needed to determine the place of rosuvastatin and other statins in the therapy of SSc and to find the right agent capable of improving cardiovascular outcome in these patients.

## Key messages

• Accelerated atherosclerosis and vasculopathy have been associated with systemic sclerosis.

• Statins may have beneficial effects on vascular function in SSc.

• In our study, rosuvastatin improved endothelial function (FMD) and decreased CRP levels suggesting a possible link between inflammation and vasculopathy in SSc.

• Complement 3, 4 and immune complex levels also decreased upon rosuvastatin treatment.

## Abbreviations

ABI: ankle-brachial index; ACE: angiotensin converting enzyme; ANA: antinuclear antibody; BAD: brachial artery diameter; BMI: body mass index; ccIMT: common carotid intima-media thickness; C3, C4: complement 3 and 4 factors; CRP: C-reactive protein; CV: coefficient of variation; ECG: electrocardiogram; EF: ejection fraction; ENA: extractable nuclear antigen; eNOS: endothelial NO synthase; ESR: erythrocyte sedimentation rate; FMD: flow-mediated vasodilation; HDL: high density lipoprotein; IC: immune complex; ICAM-1: intercellular adhesion molecule 1; ICC: intraclass coefficient of correlation; IMT: intima-media thickness; LD: laser doppler; LDL: low density lipoprotein; PAH: pulmonary arterial hypertension; PORH: post-occlusive reactive hyperemia; PU: perfusion unit; PWV: pulse-wave velocity; RNP: ribonucleoprotein; Sm: Smith antigen; SSc: systemic sclerosis; TG: triglyceride; vWF: von Willebrand factor.

## Competing interests

The authors declare that they have no competing interests.

## Authors' contributions

OT was involved in patient recruitment, vascular imaging, data analysis and manuscript writing; ZS was the senior researcher involved in data analysis, manuscript writing and supervision; GK was involved in vascular imaging assessments, as well as data analysis and manuscript writing; JV participated in vascular imaging assessments and data analysis; AVO conducted laboratory assessments; GN conducted immunolaboratory assessments and data analysis; ZC conducted some vascular assessments and contributed to manuscript writing; KD was involved in patient recruitment and examination of patients including overlap patients; SS was involved in scleroderma patient recruitment and examination; ÁN conducted patient examinations and data analysis; PS supervised the vascular imaging assessment; GS was involved in patient recruitment, supervision of the whole project and manuscript preparation. All authors read and approved the final manuscript.

## References

[B1] Czirjak L Kumanovics G Varju C Nagy Z Pakozdi A Szekanecz Z Szucs G Survival and causes of death in 366 Hungarian patients with systemic sclerosis Ann Rheum Dis 2008 15 59 63 10.1136/ard.2006.066340 17519276

[B2] Czirjak L Nagy Z Szegedi G Survival analysis of 118 patients with systemic sclerosis J Intern Med 1993 15 335 337 10.1111/j.1365-2796.1993.tb00753.x 8354987

[B3] Arias-Nunez MC Llorca J Vazquez-Rodriguez TR Gomez-Acebo I Miranda-Filloy JA Martin J Gonzalez-Juanatey C Gonzalez-Gay MA Systemic sclerosis in northwestern Spain: a 19-year epidemiologic study Medicine (Baltimore) 2008 15 272 280 10.1097/MD.0b013e318189372f 18794710

[B4] Cypiene A Laucevicius A Venalis A Dadoniene J Ryliskyte L Petrulioniene Z Kovaite M Gintautas J The impact of systemic sclerosis on arterial wall stiffness parameters and endothelial function Clin Rheumatol 2008 15 1517 1522 10.1007/s10067-008-0958-1 18654732

[B5] Szucs G Timar O Szekanecz Z Der H Kerekes G Szamosi S Shoenfeld Y Szegedi G Soltesz P Endothelial dysfunction precedes atherosclerosis in systemic sclerosis--relevance for prevention of vascular complications Rheumatology (Oxford) 2007 15 759 762 10.1093/rheumatology/kel426 17244666

[B6] Lekakis J Mavrikakis M Papamichael C Papazoglou S Economou O Scotiniotis I Stamatelopoulos K Vemmos C Stamatelopoulos S Moulopoulos S Short-term estrogen administration improves abnormal endothelial function in women with systemic sclerosis and Raynaud's phenomenon Am Heart J 1998 15 905 912 10.1016/S0002-8703(98)70137-1 9812087

[B7] Bartoli F Blagojevic J Bacci M Fiori G Tempestini A Conforti ML Guiducci S Miniati I Di Chicco M Del Rosso A Perfetto F Castellani S Pignone A Cerinic MM Flow-mediated vasodilation and carotid intima-media thickness in systemic sclerosis Ann N Y Acad Sci 2007 15 283 290 10.1196/annals.1422.030 17893992

[B8] Sherer Y Cerinic MM Bartoli F Blagojevic J Conforti ML Gilburd B Ehrenfeld M Shoenfeld Y Early atherosclerosis and autoantibodies to heat-shock proteins and oxidized LDL in systemic sclerosis Ann N Y Acad Sci 2007 15 259 267 10.1196/annals.1422.028 17893991

[B9] Davignon J Beneficial cardiovascular pleiotropic effects of statins Circulation 2004 15 Suppl 1 III39 43 15198965 10.1161/01.CIR.0000131517.20177.5a

[B10] Abou-Raya A Abou-Raya S Helmii M Statins as immunomodulators in systemic sclerosis Ann N Y Acad Sci 2007 15 670 680 10.1196/annals.1423.070 17911482

[B11] Abou-Raya A Abou-Raya S Helmii M Statins: potentially useful in therapy of systemic sclerosis-related Raynaud's phenomenon and digital ulcers J Rheumatol 2008 15 1801 1808 18709692

[B12] Furukawa S Yasuda S Amengual O Horita T Atsumi T Koike T Protective effect of pravastatin on vascular endothelium in patients with systemic sclerosis: a pilot study Ann Rheum Dis 2006 15 1118 1120 10.1136/ard.2005.046870 16837498 PMC1798255

[B13] Del Papa N Cortiana M Vitali C Silvestris I Maglione W Comina DP Lucchi T Cortelezzi A Simvastatin reduces endothelial activation and damage but is partially ineffective in inducing endothelial repair in systemic sclerosis J Rheumatol 2008 15 1323 1328 18528965

[B14] Davignon J Ganz P Role of endothelial dysfunction in atherosclerosis Circulation 2004 15 Suppl 1 III27 32 15198963 10.1161/01.CIR.0000131515.03336.f8

[B15] Jones PH Davidson MH Stein EA Bays HE McKenney JM Miller E Cain VA Blasetto JW Comparison of the efficacy and safety of rosuvastatin versus atorvastatin, simvastatin, and pravastatin across doses (STELLAR* Trial) Am J Cardiol 2003 15 152 160 12860216 10.1016/s0002-9149(03)00530-7

[B16] McKenney JM Jones PH Adamczyk MA Cain VA Bryzinski BS Blasetto JW Comparison of the efficacy of rosuvastatin versus atorvastatin, simvastatin, and pravastatin in achieving lipid goals: results from the STELLAR trial Curr Med Res Opin 2003 15 689 698 10.1185/030079903125002405 14687438

[B17] Valentini G Bencivelli W Bombardieri S D'Angelo S Della Rossa A Silman AJ Black CM Czirjak L Nielsen H Vlachoyiannopoulos PG European Scleroderma Study Group to define disease activity criteria for systemic sclerosis. III. Assessment of the construct validity of the preliminary activity criteria Ann Rheum Dis 2003 15 901 903 10.1136/ard.62.9.901 12922968 PMC1754649

[B18] Preliminary criteria for the classification of systemic sclerosis (scleroderma). Subcommittee for scleroderma criteria of the American Rheumatism Association Diagnostic and Therapeutic Criteria Committee Arthritis Rheum 1980 15 581 590 10.1002/art.1780230510 7378088

[B19] Laurent S Cockcroft J Van Bortel L Boutouyrie P Giannattasio C Hayoz D Pannier B Vlachopoulos C Wilkinson I Struijker-Boudier H Expert consensus document on arterial stiffness: methodological issues and clinical applications Eur Heart J 2006 15 2588 2605 10.1093/eurheartj/ehl254 17000623

[B20] Corretti MC Anderson TJ Benjamin EJ Celermajer D Charbonneau F Creager MA Deanfield J Drexler H Gerhard-Herman M Herrington D Vallance P Vita J Vogel R International Brachial Artery Reactivity Task Force Guidelines for the ultrasound assessment of endothelial-dependent flow-mediated vasodilation of the brachial artery: a report of the International Brachial Artery Reactivity Task Force J Am Coll Cardiol 2002 15 257 265 11788217 10.1016/s0735-1097(01)01746-6

[B21] Donald AE Halcox JP Charakida M Storry C Wallace SM Cole TJ Friberg P Deanfield JE Methodological approaches to optimize reproducibility and power in clinical studies of flow-mediated dilation J Am Coll Cardiol 2008 15 1959 1964 10.1016/j.jacc.2008.02.044 18482664

[B22] Bodnar N Kerekes G Seres I Paragh G Kappelmayer J Nemethne ZG Szegedi G Shoenfeld Y Sipka S Soltesz P Szekanecz Z Szanto S Assessment of subclinical vascular disease associated with ankylosing spondylitis J Rheumatol 2011 15 723 729 10.3899/jrheum.100668 21239756

[B23] Calabia J Torguet P Garcia M Garcia I Martin N Guasch B Faur D Valles M Doppler ultrasound in the measurement of pulse wave velocity: agreement with the Complior method Cardiovasc Ultrasound 2011 15 13 10.1186/1476-7120-9-13 21496271 PMC3098145

[B24] Baguet JP Kingwell BA Dart AL Shaw J Ferrier KE Jennings GL Analysis of the regional pulse wave velocity by Doppler: methodology and reproducibility J Hum Hypertens 2003 15 407 412 10.1038/sj.jhh.1001566 12764403

[B25] Jiang B Liu B McNeill KL Chowienczyk PJ Measurement of pulse wave velocity using pulse wave Doppler ultrasound: comparison with arterial tonometry Ultrasound Med Biol 2008 15 509 512 10.1016/j.ultrasmedbio.2007.09.008 18031922

[B26] Stein JH Korcarz CE Hurst RT Lonn E Kendall CB Mohler ER Najjar SS Rembold CM Post WS Use of carotid ultrasound to identify subclinical vascular disease and evaluate cardiovascular disease risk: a consensus statement from the American Society of Echocardiography Carotid Intima-Media Thickness Task Force. Endorsed by the Society for Vascular Medicine J Am Soc Echocardiogr 2008 15 93 111 10.1016/j.echo.2007.11.011 18261694

[B27] Norgren L Hiatt WR Dormandy JA Nehler MR Harris KA Fowkes FG Bell K Caporusso J Durand-Zaleski I Komori K Lammer J Liapis C Novo S Razavi M Robbs J Schaper N Shigematsu H Sapoval M White C White J Clement D Creager M Jaff M Mohler E Rutherford RB Sheehan P Sillesen H Rosenfield K Inter-society consensus for the management of peripheral arterial disease (TASC II) Eur J Vasc Endovasc Surg 2007 15 Suppl 1 S1 75 17140820 10.1016/j.ejvs.2006.09.024

[B28] Cracowski JL Minson CT Salvat-Melis M Halliwill JR Methodological issues in the assessment of skin microvascular endothelial function in humans Trends Pharmacol Sci 2006 15 503 508 16876881 10.1016/j.tips.2006.07.008

[B29] Determinants of pulse wave velocity in healthy people and in the presence of cardiovascular risk factors: 'establishing normal and reference values' Eur Heart J 2010 15 2338 2350 20530030 10.1093/eurheartj/ehq165 PMC2948201

[B30] Conroy RM Pyorala K Fitzgerald AP Sans S Menotti A De Backer G De Bacquer D Ducimetiere P Jousilahti P Keil U Njølstad I Oganov RG Thomsen T Tunstall-Pedoe H Tverdal A Wedel H Whincup P Wilhelmsen L Graham IM SCORE project group Estimation of ten-year risk of fatal cardiovascular disease in Europe: the SCORE project Eur Heart J 2003 15 987 1003 10.1016/S0195-668X(03)00114-3 12788299

[B31] Tam LS Li E Shang Q Tomlinson B Lee V Lee K Li M Kuan WP Li T Tseung L Yip GW Freedman B Yu CM Effects of rosuvastatin on subclinical atherosclerosis and arterial stiffness in rheumatoid arthritis: a randomized controlled pilot trial Scand J Rheumatol 2011 15 411 421 10.3109/03009742.2011.586649 21867445

[B32] El-Barbary AM Hussein MS Rageh EM Hamouda HE Wagih AA Ismail RG Effect of atorvastatin on inflammation and modification of vascular risk factors in rheumatoid arthritis J Rheumatol 2011 15 229 235 10.3899/jrheum.100582 21041278

[B33] Sadik HY Moore TL Vail A Murray A Anderson M Blann A Herrick AL Lack of effect of 8 weeks atorvastatin on microvascular endothelial function in patients with systemic sclerosis Rheumatology (Oxford) 2010 15 990 996 10.1093/rheumatology/keq003 20159906

[B34] Kuwana M Okazaki Y Kaburaki J Long-term beneficial effects of statins on vascular manifestations in patients with systemic sclerosis Mod Rheumatol 2009 15 530 535 10.1007/s10165-009-0199-4 19590932

[B35] Nussinovitch U Shoenfeld Y Atherosclerosis and macrovascular involvement in systemic sclerosis: myth or reality Autoimmun Rev 2011 15 259 266 10.1016/j.autrev.2010.09.014 20863903

[B36] Rosvall M Janzon L Berglund G Engstrom G Hedblad B Incident coronary events and case fatality in relation to common carotid intima-media thickness J Intern Med 2005 15 430 437 10.1111/j.1365-2796.2005.01485.x 15836659

[B37] Lorenz MW von Kegler S Steinmetz H Markus HS Sitzer M Carotid intima-media thickening indicates a higher vascular risk across a wide age range: prospective data from the Carotid Atherosclerosis Progression Study (CAPS) Stroke 2006 15 87 92 10.1161/01.STR.0000196964.24024.ea 16339465

[B38] Nissen SE Nicholls SJ Sipahi I Libby P Raichlen JS Ballantyne CM Davignon J Erbel R Fruchart JC Tardif JC Schoenhagen P Crowe T Cain V Wolski K Goormastic M Tuzcu EM ASTEROID Investigators Effect of very high-intensity statin therapy on regression of coronary atherosclerosis: the ASTEROID trial JAMA 2006 15 1556 1565 10.1001/jama.295.13.jpc60002 16533939

[B39] Ballantyne CM Raichlen JS Nicholls SJ Erbel R Tardif JC Brener SJ Cain VA Nissen SE Effect of rosuvastatin therapy on coronary artery stenoses assessed by quantitative coronary angiography: a study to evaluate the effect of rosuvastatin on intravascular ultrasound-derived coronary atheroma burden Circulation 2008 15 2458 2466 10.1161/CIRCULATIONAHA.108.773747 18378607

[B40] Crouse JR Raichlen JS Riley WA Evans GW Palmer MK O'Leary DH Grobbee DE Bots ML Effect of rosuvastatin on progression of carotid intima-media thickness in low-risk individuals with subclinical atherosclerosis: the METEOR Trial JAMA 2007 15 1344 1353 10.1001/jama.297.12.1344 17384434

[B41] La Civita L Rossi M Vagheggini G Storino FA Credidio L Pasero G Giusti C Ferri C Microvascular involvement in systemic sclerosis: laser Doppler evaluation of reactivity to acetylcholine and sodium nitroprusside by iontophoresis Ann Rheum Dis 1998 15 52 55 10.1136/ard.57.1.52 9536825 PMC1752467

[B42] Leiter LA Rosenson RS Stein E Reckless JP Schulte KL Schleman M Miller P Palmer M Sosef F Efficacy and safety of rosuvastatin 40 mg versus atorvastatin 80 mg in high-risk patients with hypercholesterolemia: results of the POLARIS study Atherosclerosis 2007 15 e154 164 10.1016/j.atherosclerosis.2006.12.001 17229426

[B43] Peters SA Palmer MK Grobbee DE Crouse JR O'Leary DH Raichlen JS Bots ML C-reactive protein lowering with rosuvastatin in the METEOR study J Intern Med 2010 15 155 161 10.1111/j.1365-2796.2010.02230.x 20412373

[B44] Ridker PM Clinical application of C-reactive protein for cardiovascular disease detection and prevention Circulation 2003 15 363 369 10.1161/01.CIR.0000053730.47739.3C 12551853

[B45] Corrado E Rizzo M Coppola G Fattouch K Novo G Marturana I Ferrara F Novo S An update on the role of markers of inflammation in atherosclerosis J Atheroscler Thromb 2010 15 1 11 10.5551/jat.2600 20032572

[B46] Herrick AL Illingworth K Blann A Hay CR Hollis S Jayson MI Von Willebrand factor, thrombomodulin, thromboxane, beta-thromboglobulin and markers of fibrinolysis in primary Raynaud's phenomenon and systemic sclerosis Ann Rheum Dis 1996 15 122 127 10.1136/ard.55.2.122 8712862 PMC1010106

[B47] Chen M Daha MR Kallenberg CG The complement system in systemic autoimmune disease J Autoimmun 2010 15 J276 286 10.1016/j.jaut.2009.11.014 20005073

